# Reliability and stability of Bioelectronic Medicine: a critical and pedagogical perspective

**DOI:** 10.1186/s42234-025-00179-4

**Published:** 2025-07-12

**Authors:** Massimo Mariello

**Affiliations:** Department of Engineering Science, Institute of Biomedical Engineering, University of Oxford, Oxford, OX37DQ UK

**Keywords:** Bioelectronic medicine, Wearable bioelectronics, Implantable bioelectronics, Stability, Reliability, Longevity, Durability, Failure, Water permeation, Encapsulations

## Abstract

Bioelectronic Medicine relies on wearable or implantable electronic devices interfacing with the nervous system and other active tissues, offering innovative therapeutic solutions. However, the long-term reliability and stability of these devices remain critical challenges that must be addressed for widespread clinical adoption. Advances in materials science, device engineering, power management, and biocompatibility are essential to ensure sustained functionality in dynamic biological environments. This perspective highlights key factors affecting the durability, reliability and stability of Bioelectronic Medicine technologies, explores current solutions and emerging approaches, and outlines the necessary steps to achieve robust, long-lasting bioelectronic therapeutics. The personal view expressed in this article is aimed to provide structured, accessible insights that support teaching and learning, and is envisioned to help motivate other investigators to develop further strategies for achieving clinically-relevant ultra-stable bioelectronics.

## The future of Bioelectronic Medicine is soft and flexible

Bioelectronic Medicine (BM) is an emerging field that harnesses the power of miniaturized electronic devices (Table 1) to interact with the body’s electrically active tissues and organs (nervous systems, heart, muscles, etc.), offering precise, targeted treatments for a range of conditions (Koutsouras et al. 2024). Unlike traditional pharmaceuticals, which rely on chemical interactions in the bloodstream and often lead to systemic side effects, bioelectronic devices work by modulating neural or muscular activity through electrical, optical or mechanical stimulation or controlled drug delivery. This approach has the potential to revolutionize disease management, particularly for chronic conditions such as epilepsy, Parkinson’s disease, chronic pain, and autoimmune disorders. By integrating principles from neuroscience, materials science, microelectronics, and information technology, BM provides a novel therapeutic alternative that is both highly specific and adaptable to individual patient needs. In terms of specificity, BM can target precisely certain physiological functions: instead of using broad-acting drugs, BM uses electrical stimulation, sensing, or modulation to affect specific neural circuits or organs. For example, a vagus nerve stimulator can selectively target inflammatory reflex pathways without influencing unrelated systems, avoiding the off-target effects common with systemic drugs (e.g., immune suppression or gastrointestinal side effects) (Liu et al. 2024; Fitchett et al. 2021). In terms of adaptability, BM benefits from the possibility of tuning the design of the electrode arrays and the microfabricated components, as well as of having programmable and responsive systems, which allow real-time customization of stimulation parameters based on patient symptoms, physiological feedback (e.g., heart rate, cytokine levels, etc.) and lifestyle or activity level.

**Table 1:** Typical size ranges of different bioelectronic devices (Nair et al. 2023; Kim et al. 2017; Woods et al. 2020; McBeth and Achadu 2024; Singer and Robinson .^ 2021^)

**Device category**	**Sub-type/Function**	**Typical size range**	**Examples**
Implantable devices	Neural implants	10–200 µm (width) Up to ~ 5 mm (length)	Penetrating microelectrodes (Michigan probe, Utah array) for brain-machine interfaces (Choi et al. 2018)
	Cardiac pacemakers	~ 20–30 mm diameter ~ 5–10 mm thickness	Medtronic Micra (Piccini et al. 2017)
	Cochlear implants	5–10 mm (receiver) ~ 0.5 mm wide	Implanted under the skin with electrodes threaded into the cochlea
	Retinal implants	~ 1–3 mm^2^ (chip) < 100 µm thickness	Subretinal prostheses like the PRIMA implant (Muqit et al. 2023)
	Spinal cord stimulators	~ 2–10 cm (lead) < 1 mm thickness	Flexible electrode arrays to treat chronic pain or paralysis
Wearable devices	Epidermal electronics	~ 2–10 cm^2^ area < 1 mm thickness	Skin-mounted flexible circuits for sensing electrophysiological signals (e.g., ECG, EMG, etc.)
	Smart patches	2–10 cm^2^ area 0.5–2 mm thickness	Adhesive health monitors for glucose, hydration or motion
	Textile-integrated sensors	Variable (thread/fiber level)	Sensors woven into fabrics for motion or biopotential monitoring
	Wearable ECG/EOG/EEG monitors	~ 3–10 cm^2^ area ~ 0.5–1 cm thickness	Includes chest straps, headbands and smartwatches
Ingestible devices	Smart pills	~ 10–25 mm length ~ 5–15 mm diameter	PillCam (Capsules and | Medtronic (UK) (n.d.), or other smart pills for imaging or drug delivery
	Gastric sensors	< 1 cm^3^	Sensors for internal temperature, pH or pressure
Injectable devices	Soft injectable electronics	100 µm – 1 mm diameter	Injectable flexible mesh electronics
	Hydrogel-based implants	~ 0.5–3 mm	Swellable bioelectronics for drug release or tissue integration
Microelectromechanical (MEMS) sensors	Pressure, flow or strain sensors	~ 10 µm – 1 mm	MEMS-based transducers integrated into vascular or neural systems
	Electrochemical biosensors	100 nm – 500 µm	Glucose, lactate or neurotransmitter sensors using miniaturized electrodes
Nanobioelectronics	Nanowire/nanotube transistors	< 100 nm – 1 µm	Silicon nanowires for intracellular recordings or pH sensing
	Quantum dots or nanoparticle tags	< 10–50 nm	Used for bioimaging or molecular-scale electrical sensing
	DNA-based bioelectronic interfaces	Molecular scale (few nm)	Engineered to interact with biological systems at the molecular level
Hybrid/Bio-integrated systems	Self-powered systems	1–10 mm^2^	Energy harvesters using body motion, glucose fuel cells, or thermoelectrics
	Wireless power modules	~ 0.5–5 mm	Coils and antennas integrated with implants to receive RF or ultrasound power

The origins of BM can be traced back centuries, with reports from ancient civilizations using electric fish to treat migraines and other ailments (Tsoucalas et al. 2014). The field took a major leap forward in the eighteenth century when Luigi Galvani demonstrated that electrical stimulation could activate muscle movement in frogs, laying the foundation for the study of bioelectricity (Swash 2008). By the mid-twentieth century, BM entered the clinical stage with the development of implantable pacemakers in 1958 to regulate heart rhythms (Aquilina 2006), followed by cochlear implants in the 1960 s to restore hearing in patients with profound deafness (Mudry and Mills 2013). Over time, advances in technology led to the development of deep brain stimulation (DBS) for movement disorders (Gardner 2013), spinal cord stimulation (SCS) for chronic pain (Ho et al. 2024), and vagus nerve stimulation (VNS) for epilepsy and depression (Afra et al. 2021). Today, BM continues to expand, with research exploring its applications in cardiovascular diseases, metabolic disorders, and even immune system modulation.

A defining trend in recent years is the shift toward soft and flexible bioelectronics, particularly for implantable systems (Won et al. 2020). Early bioelectronic implants were made from rigid materials like silicon and metal, which, while effective, posed challenges related to mechanical mismatch with the body’s soft tissues (Carnicer-Lombarte et al. 2021). This stiffness could lead to discomfort, inflammation, fibrosis, and even device failure over time. The human body is composed of soft, dynamic, and continuously moving tissues (Guimarães et al. 2020), making it critical for implanted devices to conform and integrate seamlessly with their biological environment (Boufidis et al. 2025; Rivnay et al. 2025).

To address this challenge, researchers have developed soft and flexible bioelectronic devices using advances in materials science and microscale engineering (Someya et al. 2016). These devices can be distinguished from rigid bioelectronics in terms of several quantifiers, presenting advantages and disadvantages concerning tissue integration, failure rates and clinical adoption (Table 2). Innovations in stretchable electronics, ultrathin films, liquid metals, hydrogels, and bioresorbable materials have paved the way for next-generation implants that are biocompatible, minimally invasive, and capable of long-term operation (Zhao et al. 2024). These soft materials allow for better mechanical compliance with tissues, reducing inflammation and improving signal transmission between the device and biological structures (Lacour et al. 2016). Additionally, flexible devices can be designed to wrap around nerves, conform to the surface of organs, or be integrated into wearable patches that provide real-time monitoring and stimulation: this prevents the need for rigid enclosures on the front-end part of the devices, whereas the back-end electronics is yet typically enclosed in rigid packages (Boys 2024; Cea et al. 2023).

**Table 2:** Comparison between rigid and soft/flexible bioelectronics (Lacour et al. 2016; Sun et al. 2023; Jiao et al. 2025)

Property	Rigid bioelectronics		Soft and flexible bioelectronics	
Typical material types	Silicon, metals, ceramics		Polymers, elastomers, hydrogels. Thin-film materials, meshes, kirigami/origami designs	
Young’s modulus	> 1 GPa		1 kPa – 1 MPa (typically)	
Bending stiffness	> 10^–6^ Nm		< 10^–9^ Nm	
Device thickness	> 100 µm		< 100 µm	
Stretchability	< 1% (brittle)		> 10% (> 100% for ultra-soft devices)	
	Advantages	Disadvantages	Advantages	Disadvantages
Tissue integration	Mechanically stable in dry environments	Stiffness mismatch causes inflammation and fibrotic encapsulation	Soft, conformal materials match tissue mechanics and reduce immune response	Soft materials may delaminate or degrade in moist biological environments
Mechanical compliance	High stiffness and robustness	Brittle under strain, poor strain tolerance	Stretchable and bendable; can tolerate body movement	Prone to mechanical fatigue, especially at interconnects
Signal fidelity	Strong short-term signal quality	Long-term degradation due to micromotion and scar tissue	Better chronic signal due to stable tissue contact	Can degrade over time due to soft substrate fatigue
Miniaturization potential and manufacturability	Established mass production with well-developed CMOS microfabrication and integration methods	Less adaptable to curvilinear or dynamic surfaces, and extreme miniaturization without mechanical loss	Emerging techniques enable stretchable circuits, ultrathin formats, and injectable designs	Fabrication more complex, with lower yield and limited by material conductivity and encapsulation needs
Implantation/comfort	Rigid housing eases handling during implantation	Bulkier, may require larger surgical pockets	Conformal, soft designs improve comfort and reduce surgical invasiveness	May require new surgical techniques
Power/data interfaces	Mature, robust wired and wireless interface options	Interfaces can damage surrounding tissues or loosen over time	Can use soft antennas or skin-contact power delivery	Less efficient wireless systems; fragile connectors
Device longevity and failure rates	Encapsulated for long-term durability. Lower failure rates under static or well-protected conditions. Typical failure modes: connector fatigue, lead fracture, infection	Long-term immune rejection and encapsulation reduce performance. Higher failure rates near joints or mobile tissue	Better biocompatibility in motion-rich environments. Lower failure rates in dynamic, soft-tissue environments. Less inflammation and better chronic integration	Degradation of soft materials over months to years, due to material degradation, fluid ingress or mechanical fatigue at soft-hard interfaces
Clinical adoption	Widely used in cardiac, neuro, cochlear and stim implants	Limited evolution in adaptability and chronic tolerance	Innovative devices (e.g., skin, neural mesh) with promising trials	Fewer regulatory approvals; emerging technologies

Beyond their physical advantages, soft bioelectronics enable more dynamic and adaptive therapeutic strategies. Traditional implants deliver fixed stimulation patterns, requiring manual adjustments by physicians. In contrast, new flexible bioelectronic systems are increasingly incorporating closed-loop feedback mechanisms, where sensors embedded in the device continuously monitor physiological signals, such as neural activity, heart rate, or inflammation markers, and adjust stimulation parameters in real time (Oh et al. 2024). This capability is particularly valuable for treating neurological conditions like epilepsy, where early detection of an impending seizure could trigger a precisely timed intervention, preventing its onset.

Another important advantage of soft and flexible bioelectronics is their potential to reduce environmental impact and improve long-term cost efficiency. Unlike traditional pharmaceuticals, which require continuous manufacturing and distribution, bioelectronic implants could provide a one-time, long-term treatment with minimal maintenance. Some emerging technologies even explore the use of bioresorbable materials, which dissolve safely in the body over time, eliminating the need for surgical removal and reducing medical waste (Zhang et al. 2023). Battery-free bioelectronic devices, powered by bioenergy harvesting or wireless energy transfer, further enhance the sustainability of this approach by eliminating the need for frequent battery replacements (Mariello and Proctor 2025; Mariello et al. 2024).

Looking ahead, the future of BM lies in the convergence of advanced materials, miniaturized flexible electronics, and intelligent data-driven control systems. By embracing soft and adaptive architectures, the next generation of bioelectronic devices will not only improve patient outcomes and quality of life but also open new frontiers in personalized medicine (Sahasrabudhe et al. 2025). With continued interdisciplinary collaboration and technological innovation, BM is poised to become a fundamental pillar of 21st-century healthcare, transforming the way we diagnose, treat, and manage disease. Currently, a complete BM system consists of three components: the implant (it interacts with the tissues and it is becoming increasingly bidirectional, multimodal, capable of biosensing and drug delivery), an optional wearable counterpart (it enhances data transfer and adaptive therapy), and the user interface (it connects patients, physicians, and cloud services for personalized treatment) (Koutsouras et al. 2024).

## Reliability and stability of future BM: notions and definitions

**Fig. 1:**
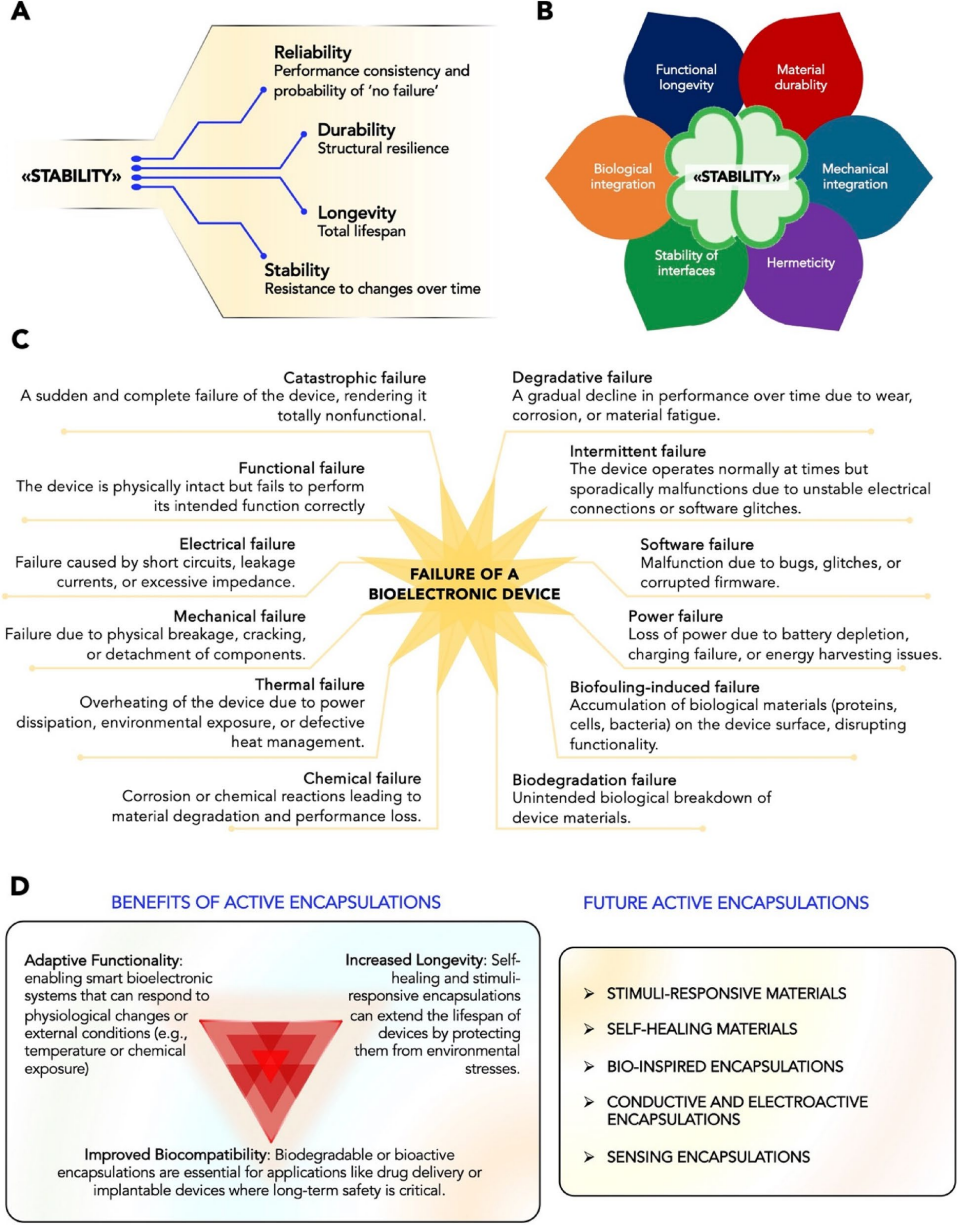
Reliability and stability of Bioelectronic Medicine: notions and concepts. **A** Generalized concept of stability of Bioelectronic Medicine, which comes in the form of reliability, durability, stability and longevity. **B** Factors affecting the stability of Bioelectronic Medicine. **C** Different failure modes of a bioelectronic device. **D** Active encapsulations: types and benefits

As BM continues to evolve, and implants become smaller and more complex, ensuring the long-term performance of implantable and wearable devices is critical for their widespread clinical adoption. This perspective aims to offer, through this and the next sections, structured and accessible insights that support teaching and learning, such as providing clear definitions of key concepts (i.e., reliability, stability, durability, and longevity, each representing distinct yet interconnected aspects of device performance (Fig. 1A) (Lanza et al. 2020)); categorizing the current strategies to achieve reliable long-term operation, and highlighting interdisciplinary connections.

Reliability refers to the probability that a bioelectronic device will function as intended, i.e., consistently and accurately, without failure over a specified period and under expected operating conditions. It is typically quantified using metrics such as failure rates, mean time between failures (MTBF), and probability of failure. It is otherwise defined as the time that one electronic device can continuously operate in a predefined operation window, without exceeding acceptable failure levels (Lanza et al. 2020; IPC 9701A-2006 - Performance Test Methods and Qualification Requirements for Surface Mount Solder Attachments n.d.).

Stability, on the other hand, denotes the ability of the device to maintain its functional and structural properties over time, including resistance to environmental and biological fluctuations. Stability can refer to electrical, chemical, or mechanical properties remaining unchanged within acceptable limits during operation. Stability refers to the degradation of the properties of the devices with time, even if unrelated to operational stresses, but instead by contamination produced by e.g., the relative humidity of the environment and/or atomic diffusion. A stable device exhibits minimal drift in performance, ensuring predictable outcomes for the patient.

Durability pertains to the device’s physical (structural) resilience and robustness, representing its ability to withstand external stresses such as mechanical deformation, temperature fluctuations, and exposure to bodily fluids without compromising function or with no significant degradation.

Finally, longevity defines the total operational lifespan of the bioelectronic device before it becomes nonfunctional or requires replacement or intervention. While a durable device can resist wear and tear, its longevity is ultimately determined by factors such as material degradation, power supply limitations, and biological interactions.

To illustrate these concepts, let us consider examples of devices that demonstrate these characteristics in varying degrees (Table 3).

**Table 3:** Examples of bioelectronic devices used in medical applications, clarifying the concepts described in this work, i.e., reliability, stability, durability and longevity

Case	Device description	Reliable	Stable	Durable	Longevous
I	A neurostimulator that functions consistently within a stable operating range, but fails intermittingly due to occasional failure in signal transmission	**-**	** × **	**-**	**-**
II	A disposable ECG patch that provides accurate signal acquisition for heart rate monitoring over a short period, but suffers from degradation of its adhesive materials due to sweat, moisture, or skin oils	** × **	**-**	**-**	**-**
III	A pacemaker constructed with highly durable materials but whose internal components degrade over time due to corrosion from bodily fluids or mechanical wear from the constant movement of heart. This can give fluctuations in its electrical output and unstable performance	**-**	**-**	** × **	** × **
IV	A wearable glucose monitor, made of durable materials, that consistently gives accurate readings over time under the same conditions, but is prone to fluctuations in its sensor readings when it is exposed to heat, humidity, or sweat	**-**	** × **	** × **	** × **
V	A deep brain stimulator, with a long operational lifespan, thanks to a robust power management system and durable materials, but with an unstable electrical stimulation due to gradual degradation of the electrode-tissue interface or biofouling	**-**	**-**	** × **	** × **
VI	A disposable smart contact lens for glucose monitoring and intraocular pressure monitoring, that provides consistent and accurate measurements, and is made of durable materials	** × **	** × **	** × **	**-**
VII	An implantable cardiac defibrillator built to withstand the harsh physiological environment of the heart, that functions consistently over time and maintains stable delivery of shocks as needed. However, the device is powered by a battery, so it requires replacement	** × **	** × **	** × **	**-**

Case I (Stable, but Unreliable). A neurostimulator that functions consistently within a stable operating range (i.e., the stimulation remains constant over time) may not be entirely reliable due to occasional failure in signal transmission. For instance, it might occasionally lose its power source or experience internal circuit malfunctions. In this case, the device operates predictably when it works but fails intermittently, making it unreliable even though it remains stable in its normal performance.

Case II (Reliable, but non-Durable). A disposable electrocardiogram (ECG) patch can provide highly reliable heart rate monitoring with accurate signal acquisition over a short period. However, it is not durable because its adhesive material degrades quickly due to sweat, moisture, or skin oils, limiting its structural integrity and reusability.

Case III (Durable, Longevous, but Unreliable). A pacemaker can be constructed with highly durable materials such as titanium, enabling it to resist physical damage, wear, and external environmental stress. However, if the pacemaker’s internal components (such as electrodes, interconnects, electronic chips) degrade over time due to the biological environment, such as corrosion from bodily fluids or mechanical wear from the constant movement of the heart, its performance may not be stable. In this example, the device may last a long time without being physically damaged (durable), but the fluctuations in its electrical output due to degradation could lead to instability in performance.

Case IV (Durable, Stable, but Unreliable). A wearable glucose monitor that consistently gives accurate readings over time under the same conditions, ensuring its reliability. However, if the device is prone to fluctuations in its sensor readings when it is exposed to heat, humidity, or sweat (which can alter its sensor properties), it will be considered unreliable in those circumstances, despite being otherwise dependable in stable environments. Thus, while it works as expected under normal conditions, its stability could suffer under varying environmental factors.

Case V (Durable, Longevous, but Unstable). A bioelectronic implant, such as a deep brain stimulator, may have a long operational lifespan (longevity), thanks to a robust power management system and materials that resist wear and tear over many years. However, the implant could lose its stability if its electrical stimulation changes slowly over time due to gradual degradation of the electrode-tissue interface or biofouling. In this case, the device would be able to last for a long time, but it may not maintain consistent therapeutic outcomes throughout its lifetime.

Case VI (Durable, Reliable, but non-Longevous). A disposable smart contact lens for glucose monitoring and intraocular pressure monitoring can be reliable, providing consistent and accurate measurements, and durable enough to resist minor scratches or moisture exposure. However, it is not long-lasting because it is designed for short-term use (e.g., a single day or a few weeks) before needing replacement.

Case VII (Reliable, Durable, Stable, but non-Longevous). An implantable cardiac defibrillator is built to withstand the harsh physiological environment of the heart (durability), function consistently over time (reliability), and maintain stable delivery of shocks as needed (stability). However, this device is often powered by a battery, and its longevity may be limited by the battery’s lifespan. Once the battery is depleted, the device will require replacement, despite having been reliable, durable, and stable during its operational period.

These examples highlight the nuanced ways in which these four concepts, reliability, stability, durability, and longevity, interact in bioelectronic devices. Understanding these differences is crucial for the development of future BM, as each device must be designed to optimize these characteristics depending on its intended application and the challenges presented by the human body. For example, a reliable device might be able to deliver consistent therapy, but if it lacks stability in certain environments, it could lead to suboptimal outcomes. Similarly, a device that is durable and resistant to mechanical wear but fails to maintain stability over time will not be able to provide effective long-term therapy. The generalized concept of “stability”, as known in the scientific community, encompasses all the previous definitions and is affected by several factors, i.e., functional longevity, material durability, biological integrability, stability of interfaces, hermeticity, mechanical integrability. Figure 1B illustrates the concept of “stability” of BM as a four-leaf clover: achieving the positive combination of all the aforementioned factors is challenging, especially when dealing with the development of new bioelectronic materials and device architectures/designs.

A crucial aspect of BM is understanding the failure modes of these devices, as failures can occur due to various electrical, mechanical, chemical, and biological factors (Fig. 1C) (Dalrymple et al. 2025; Li et al. 2021; Ohring et al. 2015). Electrical failures are among the most critical, as they can disrupt the therapeutic function of the device. These failures may arise from circuit degradation, insulation breakdown, electrode corrosion, or power supply depletion. Over time, exposure to the physiological environment can lead to changes in electrode impedance, reducing the efficiency of signal transmission and potentially leading to loss of function (Fallegger et al. 2019; Schiavone et al. 2020). Mechanical failures occur when the physical structure of the device is compromised. Implanted bioelectronics are subjected to constant motion, pressure, and strain within the body, which can cause fractures, delamination, or material fatigue in rigid components (Niemiec and Kim 2023). This is particularly relevant for older-generation implants that rely on stiff materials, leading to potential discomfort, migration, or breakage. Chemical failures involve degradation due to interactions with the biological environment. Devices must withstand prolonged exposure to bodily fluids, which contain enzymes, ions, and reactive oxygen species that can corrode materials, degrade insulation layers, or alter the performance of electronic components (Mariello et al. 2022). Encapsulation strategies, such as atomic layer deposition (ALD) and multilayer polymer coatings, are crucial in mitigating these effects by creating protective barriers against moisture and ion penetration (Mariello et al. 2024). Lastly, biological failures arise from immune responses, biofouling, or tissue reactions that affect device function. The formation of fibrotic tissue around an implant can impede electrical communication, while bacterial colonization can lead to infections and necessitate device removal (Barone et al. 2022).

Future BM must integrate innovative materials and design strategies to enhance reliability, stability, durability, and longevity, addressing these failure mechanisms proactively, and satisfying some representative benchmarks and performance thresholds (Table 4) (Oldroyd and Malliaras 2022). Advances in soft and flexible electronics, self-healing materials, bioresorbable substrates, and real-time diagnostic feedback systems will be pivotal in ensuring that bioelectronic devices remain effective, safe, and long-lasting. By adopting a holistic approach that considers both engineering challenges and biological interactions, the next generation of BM can achieve robust and sustainable therapeutic solutions.

**Table 4:** Representative quantitative benchmarks and performance thresholds related to reliability, stability, durability and longevity of bioelectronic devices (Mariello et al. 2022; Oldroyd and Malliaras 2022; Oldroyd et al. 2023; Hauser et al. 2021; Schulte et al. 2024; Jeong et al. 2020; Leterrier 2003; Mariello et al. 2024; Nisato et al. 2001; Caldwell et al. 2020; Cella 1992)

**Performance metric**	**Benchmark value, performance threshold**	**Notes**
Reliability Related to the probability of failure-free operation over time	> 95% over 1 year (clinical-grade implants) > 99% for < 1 week (disposables)	Varies by use case: high for pacemakers, lower for short-term sensors
Durability Resistance to mechanical, electrical and chemical fatigue	> 10^6^ flexural cycles (wearables) > 10^8^ cycles (stretchable ICs) > 30 MPa tensile stress (flexible traces)	Often tested with dynamic loading, stretching, bending or immersion in simulated body fluid
Stability (electrical) Retention of signal amplitude, signal-to-noise ratio (SNR), impedance (Z), gain or bandwidth, over time	< 10% drift over 1 month Z < 100 kΩ (chronic electrodes) ΔZ < 20% over 6 months SNR > 10 dB (neural) Bandwidth > 1 kHz	Important for neural or cardiac sensing. High-quality chronic interfaces aim for consistent electrical characteristics
Stability (biological) Resistance to degradation in physiological environments, over time	> 6 months (for chronic implants) > 1 year (FDA-approved devices)	Measured by encapsulation integrity, polymer stability, corrosion resistance
Stability (thermal) Temperature tolerance without performance loss, over time	−20ºC to 85ºC (rigid medical electronics) 0ºC to 45ºC (skin-worn sensors)	Important for sterilization and daily operation
Stability (mechanical) Retention of mechanical properties (modulus, elongation, toughness, etc.) over time	< 70% modulus retention after 3–6 months in PBS or sweat (Parylene, polyimide)	Mechanical degradation depends on fluid exposure, oxidative conditions and temperature
Longevity (implants) Total functional lifetime inside the body	6 months – 2 years (research-grade flexible implants) 5–15 years (rigid cardiac/neurological implants)	FDA-grade implantables are required to meet multi-year longevity
Longevity (wearables) Operational time span under daily use	3 days – 2 weeks (single-use sensors) > 6 months (reusable wearables)	Affected by battery life, skin adhesion, waterproofing
Packaging integrity Effectiveness of encapsulation against biofluid ingress	< 10^–9^ cm^3^/s He leak rate (implants) < 10^–6^ g/m^2^/day Mg test water transmission rate (implants) < 10^–6^ g/m^2^/day Ca test water vapour transmission rate (encapsulations) > 1–2% crack onset strain (encapsulations)	Standard: ISO 10993, MIL-STD-883 for hermetic packaging

## Packaging solutions in MedTech companies

Packaging solutions for bioelectronic devices are critical in ensuring the functionality, safety, and longevity of these devices when implanted or used in the human body (Mariello et al. 2022; Sun et al. 2025). As these devices interface directly with biological tissues, the packaging must protect the delicate internal components, ensure long-term stability, and minimize the risk of adverse biological reactions. Packaging solutions generally fall into three main categories, currently adopted by MedTech companies (Table 5): rigid metal or ceramic cans, soft silicones (both as substrates and encapsulations), and thin-film encapsulations (TFE). Each category offers distinct advantages and limitations depending on the specific use case and the required mechanical and biological properties.

**Table 5:** Examples of MedTech companies using different packaging strategies. RC: rigid can; SE: soft encapsulation; TFE: thin-film encapsulation

MedTech company	Device/Application field	Encapsulation Strategy		Refs
Medtronic	Percept PC, InterStim/ Deep brain stimulation, sacral neuromodulation	RC	Titanium cans + epoxy feedthroughs	Liberman and Valiquette (2011)
Medtronic	SynchroMed II/Intrathecal drug delivery pump for pain and spasticity	RC + SE	Titanium case + polymeric catheters	Medtronic (n.d.)
Boston Scientific	Vercise DBS, Spectra SCS/Brain and spinal cord stimulation	RC	Titanium can + polymer lead encapsulants	Vercise and System - Boston Scientific (n.d.)
NeuroPace	RNS System (responsive neurostimulation)/ Epilepsy treatment with a skull-mounted closed-loop device	RC	Titanium + hermetic feedthrough	The RNS System | NeuroPace (n.d.)
SetPoint Medical	Vagus nerve stimulator/Inflammatory diseases	RC + SE	Titanium enclosure + silicone header	SetPoint Medical (n.d.)
Second Sight	Argus II Retinal Implant/Vision restoration	SE + TFE	Polyimide + platinum + silicone	Farvardin et al. (2018)
Synchron	Stentrode/Brain-computer interface (BCI)	TFE	Stent-mounted; metal + polymer insulation	Mitchell et al. (2023)
Neuralink	Brain–machine interface (N1 chip)/BCI, neural prosthetics: threadlike electrodes with embedded ASIC	RC	Metal can + Parylene/polyimide thin-film	Musk (2019)
Precision Neuroscience	Layer 7 Cortical Interface/Minimally invasive BCI: skull-surface, ultrathin high-density neural array	TFE	Polymer thin-film + conformal silicone	Precision (n.d.)
Cala Health	Cala Trio/Non-invasive wearable neuromodulation for treating essential tremor	SE	Medical-grade silicone enclosure	Husaini et al. (2023)
BioInduction/Amber Therapeutics	Picostim/Cranially mounted system for deep brain stimulation	RC	Miniaturized titanium can	Zamora et al. (2022)
Salvia BioElectronics	Flexible neurostimulator/Conformal subcutaneous implant for treating chronic migraine	TFE + SE	Parylene C + PDMS hybrid	Salvia BioElectronics (n.d.)
Axonics	Sacral neuromodulation implant/Rechargeable neurostimulator for treating urinary incontinence	RC + SE	Hermetic titanium case + soft leads	Sacral Neuromodulation Therapy Treatment Details (n.d.)
Valencia Technologies	eCoin/ Wireless subcutaneous implant for tibial nerve stimulation	RC	Mini titanium capsule	Valencia Technologies (n.d.)
Vivonics/Microchips Biotech/DaréBio	Microchip drug pump/MEMS-based programmable release system	RC	Hermetic ceramic/titanium	Farra et al. (2012)
Galvani Bioelectronics	Peripheral neuromodulation/Metabolic, immune modulation, glucose and inflammatory nerve control	RC	Titanium + flexible leads	Galvani Bioelectronics (n.d.)
Pixium Vision	Prima retinal implant/Wireless subretinal implant for vision restoration	TFE	Flexible polyimide arrays + microcapsule	Muqit et al. (2023)
BIOTRONIK	Cardiac, neurostimulators/Pacemakers, neuro devices	RC	Hermetic cans + soft leads	Wollmann et al. (2012)
Neuspera Medical	Wireless neuromodulation/Peripheral stimulation	TFE	Mini capsule + flexible leads	Mid-Field Powering Technology | Sacral Neuromodulation | OAB Relief | Neuspera (n.d.)
Theranica	Nerivio/Migraine therapy	SE	Medical-grade silicone	Migraine Headache Treatment | Theranica (n.d.)
Panaxium	Organic bioelectronics/Fully soft, organic, stretchable electronics for neural sensing/stimulation	TFE + SE	PEDOT:PSS + elastomers	Panaxium (n.d.); Proctor et al. (2019)
Cortec Neuro	UtECoG, Micro Cuff, Depth Arrays/ Cortical and peripheral interfacing	SE	Silicone + metal foils	°AirRay Grid and Strip Electrodes by CorTec | Interconnection to the Central Nervous System. CorTec | Thinking ahead – Innovation in Neurotechnology (n.d.)
Neurosoft Bioelectronics	ECoG and depth arrays/Soft, conformable, implantable systems for high-resolution interfaces, for central and peripheral nerve recording	SE	Soft silicone + stretchable gold thin-films	Innovative Brain Computer Interfaces (BCI): SOFT ECoGTM, MINDZTM, SOFT TINNITTM, and Synapsuit. Neurosoft Bioelectronics (n.d.)
InBrain Neuroelectronics	Graphene neural interfaces/Precision neuromodulation	TFE	Parylene/graphene + flexible substrate	Ria et al. (2025)
Paradromics	Connexus Direct Data Interface/High-bandwidth brain–computer interface to translate massive neural data	RC	Metal + polymer-insulated high-density array	Connexus Brain-Computer Interface | The Future of BCI | Paradromics (n.d.)

The first category of packaging is rigid metal or ceramic cans, which are often used in implantable devices that require high protection from physical damage, biological fluids, and external forces. These cans are typically made from biocompatible materials such as titanium or ceramic and provide a robust solution for protecting electronic components. Medtronic, a leader in medical technology, uses titanium cans in some of its infusion pumps and cardiac rhythm management devices (Medtronic n.d.). These materials offer excellent mechanical strength, corrosion resistance, and biocompatibility. For instance, Medtronic’s infusion pumps, which deliver precise amounts of medication, rely on these rigid casings to protect delicate internal systems while ensuring safe long-term implantation within the body. Paradromics, a company specializing in high-bandwidth neural interfaces, also uses rigid metal cans to encase its devices (Connexus Brain-Computer Interface | The Future of BCI n.d.). These cans protect the sophisticated electronics in their cortical communication systems, offering a high degree of durability and shielding from biological factors like moisture and ionic contamination.

The second category includes soft silicones, which are increasingly used as both substrates and encapsulations due to their flexibility, biocompatibility, and ability to form intimate contact with tissues. Soft silicones are often used in wearable and implantable devices that need to adapt to the body’s natural movements. Neurosoft Bioelectronics, for example, leverages soft silicone materials in their neurostimulation devices (Innovative Brain Computer Interfaces (BCI): SOFT ECoGTM, MINDZTM, SOFT TINNITTM, and Synapsuit n.d.). These materials provide a soft, flexible base that conforms to the tissue without causing irritation or discomfort. CorTec, which specializes in minimally-invasive brain stimulation systems, also utilizes soft silicone-based encapsulations to ensure the protection of electrodes while maintaining flexibility and comfort for the patient (°AirRay Grid and Strip Electrodes by CorTec | Interconnection to the Central Nervous System. CorTec | Thinking ahead – Innovation in Neurotechnology, n.d.). Silicone substrates can also be used in combination with advanced coatings to improve the device's resistance to biological degradation, thus extending its functionality and minimizing failure rates over time. These soft materials offer a balance between performance and comfort, making them an ideal solution for many bioelectronic devices, especially those that are in constant contact with biological tissue.

Finally, TFE represent an advanced packaging solution for bioelectronics that provides high protection with minimal weight and size. Thin films are typically composed of multi-layered materials that can shield electronic components from moisture, ionic contamination, and mechanical damage. The use of thin films is common in devices that require miniaturization, such as Inbrain Neuroelectronics, which uses polyimide and graphene thin films to encapsulate their neural interfaces (Ria et al. 2025). This combination of materials provides excellent flexibility, electrical insulation, and mechanical integrity, ensuring that sensitive electronics can maintain stable performance over long periods within the body. The primary benefit of TFE is the ability to maintain the small form factor required for implantable devices while protecting against corrosion and physical wear. Polyimide and graphene-based thin films offer added advantages such as high thermal stability and electrical conductivity, essential for the proper functioning of bioelectronic devices in dynamic biological environments. Precision Neuroscience is also making strides in the field by adopting thin-film technology to integrate neurostimulation and bioelectronic interfaces with high precision (Precision n.d.). These technologies, combined with the advantages of soft silicones and rigid materials, allow for both reliable performance and adaptability in clinical applications.

Each packaging solution, whether rigid, soft, or thin-film, has its place in the field of bioelectronics, and the choice of material depends on the specific requirements of the device, including biocompatibility, flexibility, strength, and longevity. As BM advances, these packaging technologies will continue to evolve to meet the growing demand for safe, effective, and sustainable implantable devices.

## Materials for TFE in BM

TFE are a critical component in BM, as they protect sensitive electronic components from bodily fluids while maintaining long-term functionality and biocompatibility. Traditional materials used in TFE include metals, ceramics, and organic polymers, each providing a balance of mechanical strength, flexibility, and resistance to corrosion. Metals such as titanium and stainless steel have long been used for their strength and hermetic properties, making them ideal for rigid, implantable devices. However, they often lack the flexibility needed for softer, more flexible bioelectronic devices that conform to the body’s movements. On the other hand, organic materials like silicone and polyimide are increasingly popular for their flexibility and biocompatibility. These materials can be molded to fit various shapes, offering a more adaptable approach for implants that must accommodate dynamic biological environments. Polyimide, for instance, is widely used in flexible electronics and has shown promise as a material for encapsulating bioelectronic systems, as it offers excellent compatibility with microfabrication processes, thermal stability, and electrical insulation (Mariello et al. 2024), although its relatively high Young’s modulus can induce strain at the tissue interface (Antanavičiūtė et al. 2018). On the other hand, silicones offer better softness, but they their limited patterning resolution and the need for insertion aids or carriers, makes them challenging to process and handle at micrometer scale.

Future directions for TFE in BM are likely to center around enhancing the biocompatibility and long-term stability of materials while reducing environmental impact (Mariello et al. 2022). One promising area is the integration of smart polymers or biohybrid materials, which can dynamically respond to changes in their environment. For example, polymers that change their properties in response to pH, temperature, or other biomarkers could help improve the adaptability and performance of bioelectronic implants in real-time (Martinelli et al. 2024). Another exciting avenue is the development of multi-functional materials that not only encapsulate the device but also actively support the device’s function (Mariello et al. 2021). Conductive polymers (e.g., PEDOT:PSS) (Donahue et al. 2020), which can simultaneously provide mechanical support and facilitate electrical communication with tissues, are gaining traction, although it has been well demonstrated that they suffer from in vivo degradation and oxidative instability, especially under chronic stimulation conditions (Doshi et al. 2025).

Materials like graphene and MXenes, with their exceptional electrical conductivity and mechanical flexibility, are being explored for next-generation bioelectronic interfaces, potentially enhancing both the performance and longevity of implants (Driscoll et al. 2021).

Moreover, advancements in nano-coatings and layered structures with inorganic thin-films (e.g., Al_2_O_3_, Si_3_N_4_, SiO_2_, TiO_2_, SiC) deposited by advanced techniques (e.g., chemical vapour deposition (CVD), physical vapour deposition (PVD), atomic layer deposition (ALD), thermal growth, molecular layer deposition (MLD), pulsed laser deposition (PLD), etc.), are expected to enhance the hermeticity of TFEs, providing more robust protection against environmental factors (Mariello et al. 2024). These coatings could also integrate sensors that monitor the condition of the device, providing real-time feedback to ensure that implants remain functional and safe throughout their life cycle.

One main challenge of ultrathin encapsulations is the mechanical stability of their bi-layer configuration with soft elastomeric substrates. In fact, TFE have moduli in the GPa range, while silicones have moduli in the kPa-MPa range, leading to significant strain concentration at the interface during bending or stretching. This may cause poor adhesion and delamination risks especially under cyclic mechanical stress or in a hydrated biological environment, which is particularly problematic for long-term implants where encapsulation integrity is essential for reliability. Furthermore, the high temperatures often required for CVD/ALD deposition can degrade soft polymers or introduce thermal stress that further weakens adhesion and promotes film buckling or cracking.

In the long term, the development of self-healing materials, which could repair damage caused by mechanical stress or corrosion, might revolutionize the reliability and stability of bioelectronic systems, extending their lifespan and reducing the need for invasive re-implantations (Liu et al. 2024).

In overall, biocompatible materials are essential for the long-term success of implantable bioelectronic devices, as they directly influence host-tissue interactions and the risk of chronic immune responses. Even when materials are classified as biocompatible, such as medical-grade silicone, polyimide, parylene C, or metals like platinum and titanium, they may still provoke a foreign body response (FBR) when implanted long-term, involving protein adsorption, macrophage activation, and eventually the formation of a fibrotic capsule around the implant (Carnicer-Lombarte et al. 2021; Barone et al. 2022). Over time, this encapsulation can increase impedance, reduce electrical coupling, and mechanically isolate the device from target tissues, undermining both the efficacy and stability of neural or muscular interfaces. In extreme cases, chronic inflammation may lead to tissue damage or device migration. The severity of the FBR depends on multiple factors, including the surface chemistry, topography, stiffness, and geometry of the implant. Soft and compliant materials that better match the mechanical properties of tissue can help reduce immune activation, but they must also maintain structural and functional stability over years in vivo. Therefore, designing long-term implants requires a balance between biocompatibility and durability.

## Stability of BM and hermeticity of TFE: evaluation methods

Hermeticity refers to the ability of a material or structure to maintain a complete and continuous seal, preventing the ingress of moisture, gases, or biological contaminants. In the context of BM, particularly implantable devices, hermeticity is crucial for ensuring long-term stability and functionality. These devices, often made from advanced TFE, must remain sealed for extended periods within the body to avoid failures caused by environmental factors such as bodily fluids or bacterial invasion. The hermeticity of TFE plays a pivotal role in determining the longevity and stability of bioelectronic devices, directly influencing their performance and reliability. Over time, the performance of these devices can degrade due to factors like water absorption, ionic corrosion, or mechanical stress, making the evaluation of hermeticity essential to predict their lifespan and minimize device failure.

Several methods exist to characterize the hermeticity of advanced TFEs, which can then be correlated with the stability and longevity of bioelectronic devices (Mariello et al. 2022). Direct diffusion methods involve measuring the permeability of the thin-film material to gases, such as water vapor or oxygen. This is typically achieved by exposing the encapsulated device to controlled environmental conditions and measuring the rate at which certain molecules diffuse through the material. A common example is the water vapor transmission rate (WVTR), which quantifies the amount of moisture that passes through the encapsulation. This method provides direct insight into how well the thin-film encapsulation protects the device from external contaminants, offering a reliable assessment of its hermetic properties.

Indirect electrical methods are another popular approach for evaluating hermeticity. These techniques assess the electrical behavior of the device or the encapsulating material when exposed to various environmental stresses. Changes in electrical resistance or capacitance can indicate the onset of leakage or material degradation. For example, if the encapsulation begins to lose its integrity and allows moisture to penetrate, the device’s electrical characteristics may change, providing an early warning of potential failure. These methods are often used for in-situ monitoring during device operation, offering real-time feedback on hermeticity. The most recent method in this category is the Magnesium test, demonstrated in vitro and in vivo (Mariello et al. 2024).

Indirect electrochemical methods focus on measuring changes in electrochemical behavior, such as the impedance or current flow through the thin-film encapsulation, in response to environmental conditions (Schiavone et al. 2020; Oldroyd et al. 2023). Electrochemical techniques can help identify the presence of ions or conductive pathways formed due to degradation of the material over time. This method is particularly useful for evaluating materials that are prone to corrosion, as it allows for the detection of subtle chemical changes that could compromise the hermetic seal.

Indirect optical methods employ techniques such as spectroscopy or light scattering to analyze the transparency and structure of the encapsulating material. For example, a shift in optical properties may indicate the formation of cracks or voids in the thin-film encapsulation. These methods can also be used to monitor the integrity of the encapsulation during the fabrication process, ensuring that the material remains intact before implantation; recently, Magnesium optrodes have been validated as ultra-sensitive sensors of water permeation (Mariello et al. 2024). Optical methods are non-destructive and can provide detailed, real-time insights into the condition of the thin-film.

Indirect electromechanical methods involve monitoring the mechanical properties of the thin-film encapsulation, such as its flexibility, stress, or strain (Niemiec and Kim 2023). By applying mechanical forces to the material and observing its response, these methods can detect the onset of microfractures or delamination that could compromise the hermetic seal. The deformation behavior under applied stresses or environmental conditions such as temperature fluctuations or moisture exposure provides valuable information about the device’s long-term reliability (Kim et al. 2019).

Lastly, indirect mechanical methods focus on the physical integrity of the encapsulating material by applying force to detect any changes in shape, elasticity, or hardness. Techniques like nanoindentation or microhardness testing can be used to assess the material’s resistance to wear, scratches, or other physical stresses. These methods help determine whether the thin-film encapsulation can maintain its protective properties under real-world conditions, such as the mechanical forces exerted on the device when implanted in the body.

By understanding how TFE materials behave in various environmental conditions, researchers can correlate their performance with the stability, longevity, and reliability of bioelectronic devices, ensuring their safety and effectiveness in medical applications.

### Regulatory frameworks for TFE

Regulatory frameworks governing the use of TFE in implantable bioelectronic devices are evolving to address the unique challenges posed by these materials and architectures (Lee et al. 2024). TFE, typically comprising polymers like parylene, polyimide, or inorganic coatings such as silicon dioxide and alumina, are critical for protecting sensitive electronics from the corrosive biological environment. However, their long-term stability, biocompatibility, and failure modes, raise specific safety concerns that regulatory bodies like the U.S. Food and Drug Administration (FDA) and the European Medicines Agency (EMA), and international standards organizations (e.g., ISO, IEC, etc.) are increasingly scrutinizing. For Class III implantable devices, which involve high-risk, long-term implantation, the regulatory path requires extensive documentation on material properties, sterilization compatibility, cytotoxicity, and accelerated aging performance, typically under ISO 10993 (biological evaluation), ISO 14708 (implantable neurostimulators) and IEC 60601 (safety testing) standards. Additionally, TFE often involve novel manufacturing techniques or soft-substrate integration, which may fall under “novel technology” classifications requiring pre-submission meetings or additional risk assessments. Manufacturers must demonstrate that the encapsulation maintains electrical and mechanical integrity over the device’s intended lifetime (often 5–10 years for chronic implants) through real-time or simulated in vivo testing. Regulatory expectations increasingly emphasize not only material biostability but also robust quality assurance and traceability in microfabrication processes, given the thin films’ sensitivity to defects. This evolving landscape necessitates early regulatory engagement during device development.

## Future of TFE in BM

The future of TFE in BM is not only about providing protection for delicate electronics but also about integrating dynamic functionalities that can actively respond to the body’s needs (Fig. 1D). One promising direction is the use of hydrogels as encapsulating materials, which can be loaded with drugs and other therapeutic agents. These hydrogels can serve as both the protective encapsulation and a drug-delivery system, releasing their contents in response to specific environmental cues, such as temperature, pH, or electrical signals. This approach would allow bioelectronic devices to not only interact with the body’s nervous system but also administer localized, controlled treatments, thereby enhancing therapeutic efficacy and reducing side effects.

Another exciting avenue for active TFE is the incorporation of electrochemical polymers, which can deform and actuate mechanically when an electrical stimulus is applied. These polymers can change shape in response to voltage changes, enabling the encapsulation itself to perform dynamic mechanical functions, such as opening, closing, or reshaping. This ability to create motion could be harnessed for a variety of applications, including in-vivo actuation for tissue manipulation or even for self-adjusting implants that adapt their shape based on the surrounding environment or therapeutic needs. For instance, a bioelectronic device with a stretchable electrochemical polymer-based encapsulation could adjust its form to better fit the surrounding tissue, improving comfort and functionality over time (Dong et al. 2024).

Such active thin-film encapsulations could lead to the development of more sophisticated, adaptive bioelectronic systems capable of continuous monitoring and real-time adjustments. These devices could, for example, sense fluctuations in biological signals and automatically modulate both their electrical output and drug delivery to optimize therapeutic effects. Additionally, these materials could be designed to integrate multiple therapeutic modalities, such as electrical stimulation, drug delivery, and mechanical actuation, within a single implant. The use of active, responsive encapsulations could significantly enhance the precision, versatility, and efficiency of BM, enabling the next generation of personalized, multifunctional therapies that adapt dynamically to the individual needs of patients.

## Conclusions

Ensuring the reliability and stability of BM is paramount for its successful clinical translation and long-term impact. However, to fully grasp the challenges, it is essential to distinguish between key concepts of stability, reliability, durability and longevity. This perspective contributes to this purpose, through a critical and pedagogical approach, providing clear and precise definitions of these concepts, categorizing packaging strategies, integrating comparative tables and highlighting interdisciplinary connections.

A critical factor influencing the BM’s long-term operation is the degradation of implantable devices in the dynamic and reactive biological environment. Advanced TFE techniques, such as ALD, PECVD, and multilayer barrier coatings, have emerged as promising solutions to enhance device longevity by providing ultrathin, highly conformal, and biocompatible protective layers. These encapsulation strategies mitigate moisture and ion infiltration, reducing failure rates and ensuring sustained therapeutic efficacy. Addressing these challenges requires a multidisciplinary effort, bringing together engineers, neuroscientists, material scientists, and clinicians to develop robust solutions. Additionally, regulatory frameworks and standardization must evolve to support the safe deployment of these technologies. Despite the hurdles ahead, the potential of BM to transform disease management is immense. With continued innovation in materials and encapsulation strategies, alongside collaborative efforts across disciplines, the vision of stable, reliable bioelectronic therapies as a standard of care is within reach.

## Data Availability

No datasets were generated or analysed during the current study.
